# Intestinal gluconeogenesis is downregulated in pediatric patients with celiac disease

**DOI:** 10.1186/s12916-022-02635-3

**Published:** 2022-11-11

**Authors:** Olof Karlson, Henrik Arnell, Audur H. Gudjonsdottir, Daniel Agardh, Åsa Torinsson Naluai

**Affiliations:** 1 Department of Laboratory Medicine, Institute of Biomedicine, Sahlgrenska Academy at the University of Gothenburg, Gothenburg, Sweden; 2 Department of Pediatric Gastroenterology, Hepatology and Nutrition, Astrid Lindgren Children’s Hospital, Karolinska University Hospital, Stockholm, Sweden; 3 Department of Women’s and Children’s Health, Karolinska Institute, Stockholm, Sweden; 4 Department of Paediatric Gastroenterology, Hepatology and Nutrition, Queen Silvia Children’s Hospital, Sahlgrenska University Hospital, Gothenburg, Sweden; 5 Department of Clinical Sciences, Unit of Celiac Disease and Diabetes, Lund University, Malmö, Sweden

**Keywords:** Celiac disease, Gluten, intestinal gluconeogenesis, Non-alcoholic fatty liver disease

## Abstract

**Background:**

Untreated celiac disease (CD) patients have increased levels of blood glutamine and a lower duodenal expression of glutaminase (GLS). Intestinal gluconeogenesis (IGN) is a process through which glutamine is turned into glucose in the small intestine, for which GLS is crucial. Animal studies suggest impaired IGN may have long-term effects on metabolic control and be associated with the development of type 2 diabetes and non-alcoholic fatty liver disease (NAFLD). The aim of this study was to thoroughly investigate IGN at the gene expression level in children with untreated celiac disease.

**Methods:**

Quantitative polymerase chain reaction (qPCR) was used to quantify the expression of 11 target genes related to IGN using the delta-delta Ct method with three reference genes (*GUSB*, *IPO8*, and *YWHAZ*) in duodenal biopsies collected from 84 children with untreated celiac disease and 58 disease controls.

**Results:**

Significantly lower expression of nine target genes involved in IGN was seen in duodenal biopsies from CD patients compared with controls: *FBP1*, *G6PC*, *GLS*, *GPT1*, *PCK1*, *PPARGC1A*, *SLC2A2*, *SLC5A1*, and *SLC6A19*. No significant difference in the expression was observed for *G6PC3* or *GOT1*.

**Conclusions:**

Children with untreated celiac disease have lower expression of genes important for IGN. Further studies are warranted to disentangle whether this is a consequence of intestinal inflammation or due to an impaired metabolic pathway shared with other chronic metabolic diseases. Impaired IGN could be a mechanism behind the increased risk of NAFLD seen in CD patients.

**Supplementary Information:**

The online version contains supplementary material available at 10.1186/s12916-022-02635-3.

## **What you need to know**

### Background

We have previously shown an association between celiac disease (CD) and amino acid metabolism including the gene glutaminase (*GLS*). This enzyme has an important role in intestinal gluconeogenesis (IGN) where the amino acid glutamine is the main substrate.

It has also been shown that patients with CD are at an increased risk of non-alcoholic fatty liver disease (NAFLD) later in life.

### Findings

All key genes involved in intestinal gluconeogenesis are downregulated at the gene expression level in the small intestine of children with untreated CD, suggesting impairment of intestinal gluconeogenesis.

### Implications for patient care

Impaired intestinal gluconeogenesis might lead to the increased risk of NAFLD seen in patients with CD as adults. Early diagnosis and treatment of CD may restore intestinal gluconeogenesis and prevent CD patients from NAFLD later in adulthood.

## Background

**Fig. 1:**
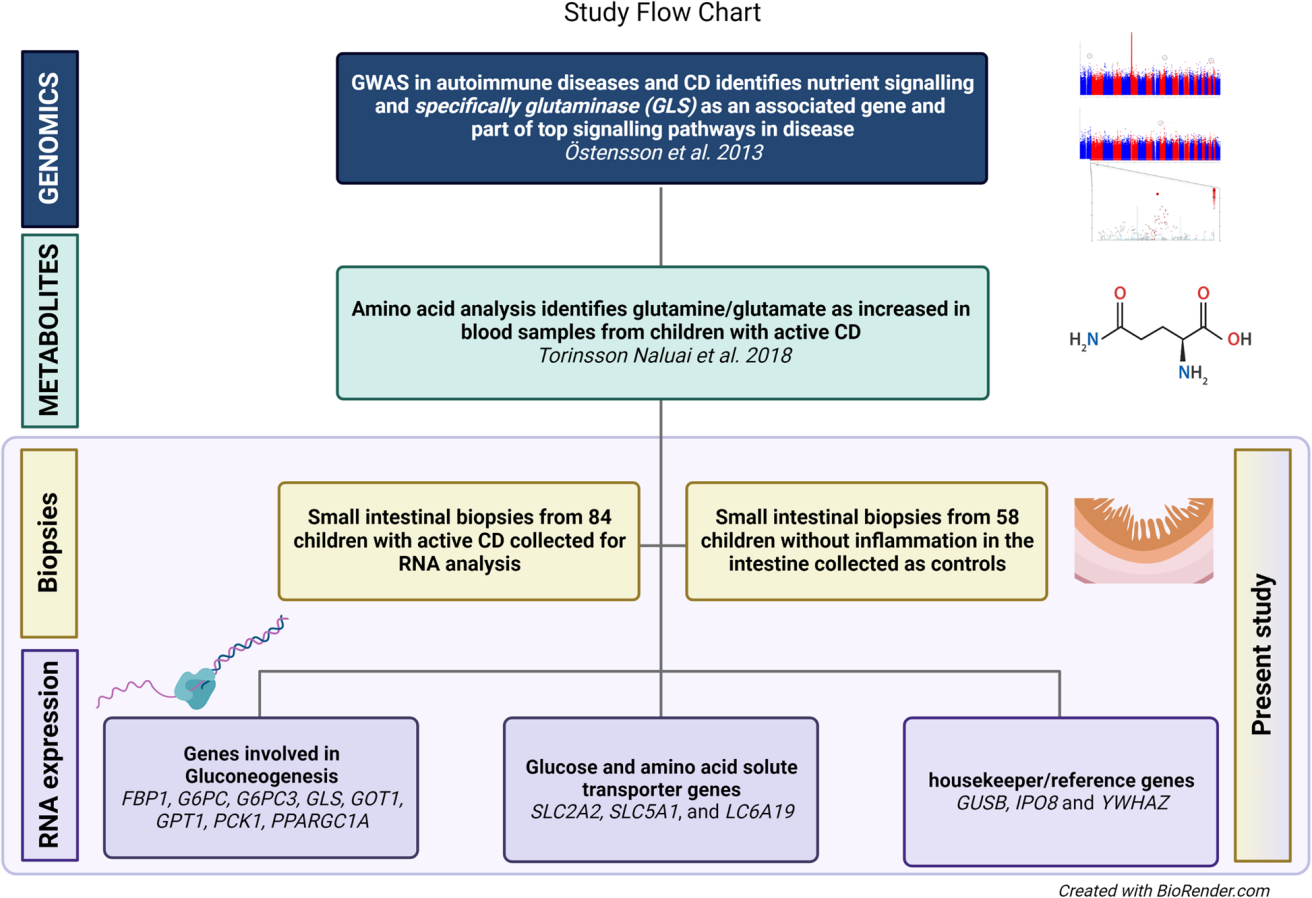
Study flow chart and outline of our previous work as well as the work in the present study

A family-based genome-wide association study (GWAS) found shared genes between celiac disease (CD) and type 2 diabetes (T2D), indicating changes in common nutrient signaling pathways of amino acid metabolism. Furthermore, glutaminase (GLS), an enzyme that converts glutamine to glutamate, was identified by pathway analysis in the most associated network of genes and was found to be downregulated in duodenal biopsies from children with untreated CD [1]. Later work showed allele-specific expression of *GLS* connected to the associated single-nucleotide polymorphism (SNP) (rs6741418) in the *GLS* gene region [2]. A total of 142 associations to 50 traits, a large number of which are autoimmune diseases, have been reported for the gene region containing *GLS/STAT1/STAT4* as reported in the GWAS Catalog*.* This makes it one of the top-reported gene regions in autoimmune diseases so far. Out of these 142 reported associations, 26 SNP variants have been shown to have a significant influence on the expression of *GLS*, while only three SNP variants influence the expression of *STAT4*, and none has been shown to influence *STAT1* expression (data from the gtexportal). Additional file 1: Table S1 shows the most associated variant reported for each trait (data from the GWAS Catalog). Thus, there is data indicating a possible common genetic mechanism behind autoimmunity and differential expression of *GLS*. Interestingly, patients with untreated CD also have altered blood levels of several amino acids, including glutamine/glutamate, further suggesting alterations of metabolism warranting more investigation [3]. Figure 1 illustrates a study flow chart and outline of previous work as well as the work in the present study.

Intestinal gluconeogenesis (IGN) is a process by which glucose can be produced in the small intestine [4]. Glutaminase has a critical role in IGN since it catalyzes the first step needed to use its main substrate, which is glutamine [5]. In animal models, IGN-deficient mice develop hyperglycemia [6]. Mice with induced high levels of IGN instead seem protected against hyperglycemia, even when fed a high-fat/high-sucrose (HF-HS) diet. IGN-deficient mice also more easily develop hepatic steatosis, both on an HF-HS diet and a standard diet, while mice with induced IGN are again protected, even on an HF-HS diet [7]. In a rat model investigating the mechanisms behind bariatric surgery as a treatment of T2D, rats showed increased IGN after surgery [8]. In human studies, T2D patients with high expression of genes involved in IGN had greater improvement in insulin resistance scores after bariatric surgery [9]. These data indicate an important role for IGN in energy homeostasis and metabolic disease, which is suggested to be explained by a glucose-sensing mechanism in the portal vein, where higher levels of glucose indicating an adequate glucose supply lead to satiety signals sent via the spinal nerves to the brain which then affects the whole-body metabolism [10].

**Fig. 2:**
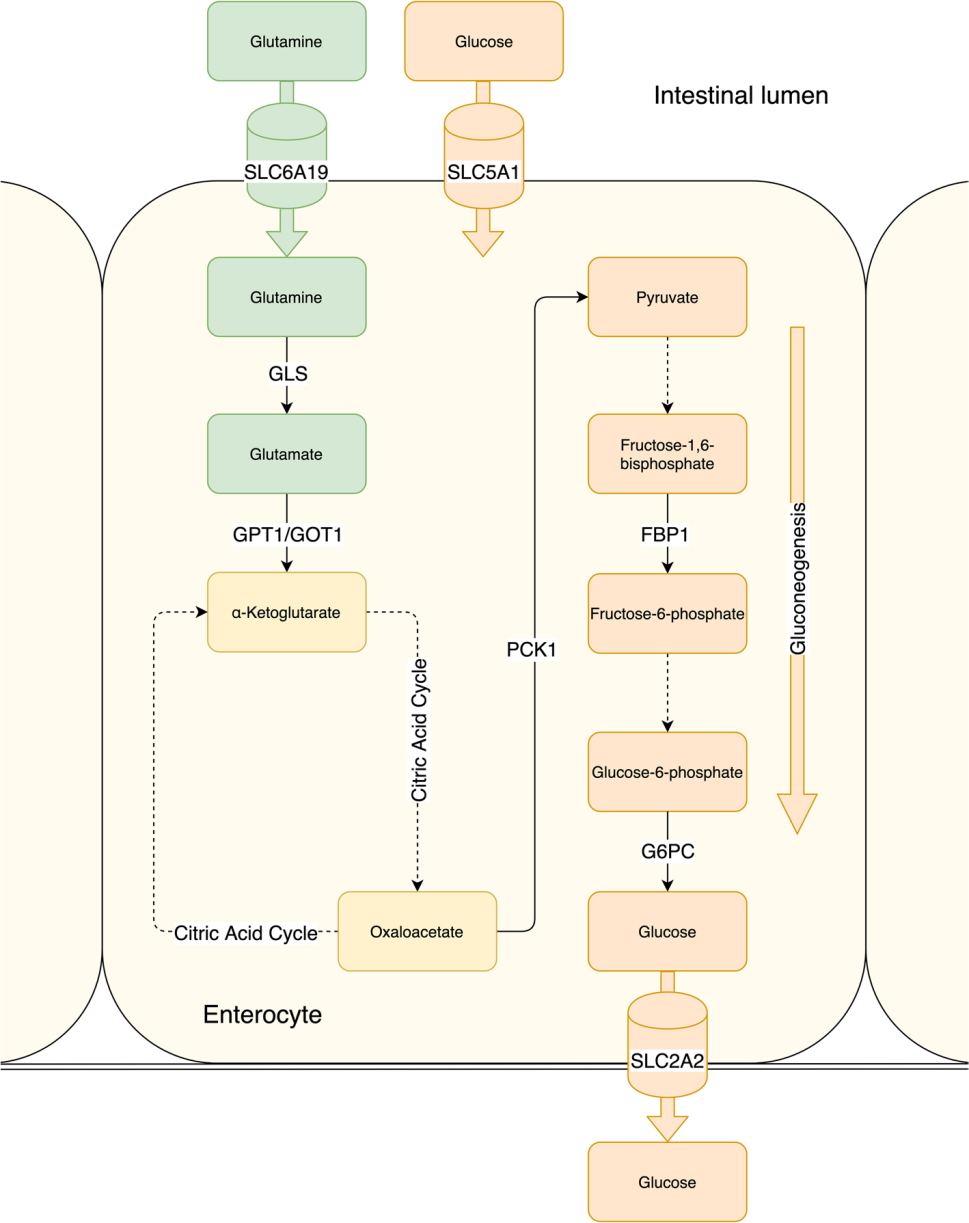
Gluconeogenesis and steps for using glutamine as a substrate for gluconeogenesis showing the enzymes for which we have quantified gene expression. Dotted lines represent steps that have been left out of the figure where gene expression has not been investigated. Investigated solute carriers are also shown

The aim of the present study was to investigate IGN in CD patients by investigating intestinal gene expression of *GLS* and another 10 selected genes involved in IGN in children with untreated CD. Figure 2 illustrates IGN and the roles of the selected genes. The hypothesis was that with *GLS* downregulated, the whole process of IGN with its important metabolic function could be affected.

## Methods

### Biological material

Duodenal biopsies were collected from children between 2010 and 2012 at four different hospitals in Sweden as previously described [11]. All children had been referred for an upper endoscopy for medical reasons and were consecutively recruited to the study, and both male and female subjects were included. For the present study, 84 untreated CD cases and 58 disease controls were used. The ESPGHAN 1999 criteria [12] were applied at all clinical sites for the clinical diagnosis of CD, e.g., a biopsy showing Marsh score > 1 at diagnosis and a clear response to a gluten-free diet with a significant reduction of IgA-tTG levels after treatment. In order to ensure unbiased classification of the cohort to cases and controls, a pathologist reviewed and scored the biopsies blinded to other clinical information [11]. Children with a Marsh score > 1 and a positive IgA-tTG serology were included as cases, and children with a Marsh score of ≤ 1 and negative IgA-tTG serology were included as disease controls (Additional file 2: Table S2). Among the included children, 64 out of 84 (76%) cases and 34 out of 58 (59%) controls were females. The mean age ± standard deviation of cases was 6.1 ± 3.7 compared with 11.6 ±4.5 years old among the controls (*p* < 0.001). Children with inflammatory bowel disease or *Helicobacter pylori* infections were excluded prior to analysis since these diseases might show inflammation in the duodenum, which in turn could influence the gene expression. No child with non-alcoholic fatty liver disease or any other liver diseases was included. All diagnoses present in the control group are listed in Additional file 2: Table S2. Duodenal biopsies were immediately put in RNA-stabilizing RNAlater solution (Life Technologies, CA, USA) and put at room temperature overnight to allow the reagent to penetrate the sample. Biopsies were then frozen at − 80 °C until RNA extraction was performed. Total RNA was extracted using the AllPrep® DNA/RNA/Protein Mini Kit (Qiagen, Germany). RNA quality and quantity were measured with a NanoDrop 2000c spectrophotometer (Thermo Fisher Scientific, MA, USA) and a 2100 Bioanalyzer (Agilent Technologies, CA, USA). RNA was converted to cDNA for storage using the SuperScript Vilo cDNA synthesis kit (Thermo Fisher Scientific, MA, USA) and stored at − 80 °C.

Children and/or parents gave their informed written consent, and the local ethical committee approved the study (T373-10).

### Quantitative polymerase chain reaction

Quantitative polymerase chain reaction (qPCR) was performed using TaqMan® technology (Thermo Fisher Scientific, MA, USA). The final reaction consisted of TaqMan® gene primers and equal parts sample cDNA and TaqMan® Universal Master Mix II. All primer-sample reactions were run in duplicate in 384-well plates on a QuantStudio 12K Flex Sequence Detection System (Thermo Fisher Scientific, MA, USA). Target genes were *FBP1* (fructose-bisphosphate 1), *G6PC* (glucose-6-phosphatase), *G6PC3*, *GLS*, *GOT1* (glutamic-oxaloacetic transaminase 1), *GPT1* (glutamic-pyruvic transaminase), *PCK1* (phosphoenolpyruvate carboxykinase 1), *PPARGC1A* (PPARG coactivator 1 alpha), *SLC2A2* (solute carrier family 2 member 2), *SLC5A1* (solute carrier family 5 member 1), and *SLC6A19* (solute carrier family 6 member 19). Reference genes were *GUSB* (glucuronidase beta), *IPO8* (importin 8), and *YWHAZ* (tyrosine 3-monooxygenase/tryptophan 5-monooxygenase activation protein zeta). These reference genes have been evaluated by the group previously using the *Normfinder* software [13].

### Statistical analysis

Quality control of qPCR data was done in the ExpressionSuite v1.1 program (Thermo Fisher Scientific, MA, USA) where reactions that had not run properly were filtered out. ΔCT values were calculated using R [14] with the RStudio developing environment (which was used for all work in R) [15]. The group differences were examined by a generalized linear model using the glm function in R, running one model based only on CD case status and one model adjusting for age and sex as covariates. A generalized linear model was used to enable adjustment for both a quantitative (age) and a qualitative variable (sex). With *a* = 0.05 correcting for multiple testing of 11 target genes using the Bonferroni method gave 0.05/11 = 0.0045 as the adjusted significance threshold. Data visualization was done using the ggplot2 package in R [16], parts of tidyverse [17] from which other packages were also used when coding in R. Fold change was calculated using the 2^−ΔΔC^ method [18]. A partial correlation analysis using gene expression levels, tissue transglutaminase (tTG) antibody levels, and Marsh score controlling for age was performed in the IBM SPSS statistics software version 27. *p*-values were set as 2-sided. Marsh scores of all cases were transformed into numbers: Marsh stage 2, 1; Marsh stage 3A, 2; Marsh stage 3B, 3; and finally, Marsh stage 3C, 4.

We used the STROBE case-control reporting guidelines when writing this paper (von Elm E, Altman DG, Egger M, Pocock SJ, Gotzsche PC, Vandenbroucke JP. The Strengthening the Reporting of Observational Studies in Epidemiology (STROBE) Statement: guidelines for reporting observational studies). All authors had access to the study data and had reviewed and approved the final manuscript.

## Results

All samples that were included passed quality control for RNA 260/230 value above 1.8 and were used for analysis.

### Gene expression levels

**Fig. 3:**
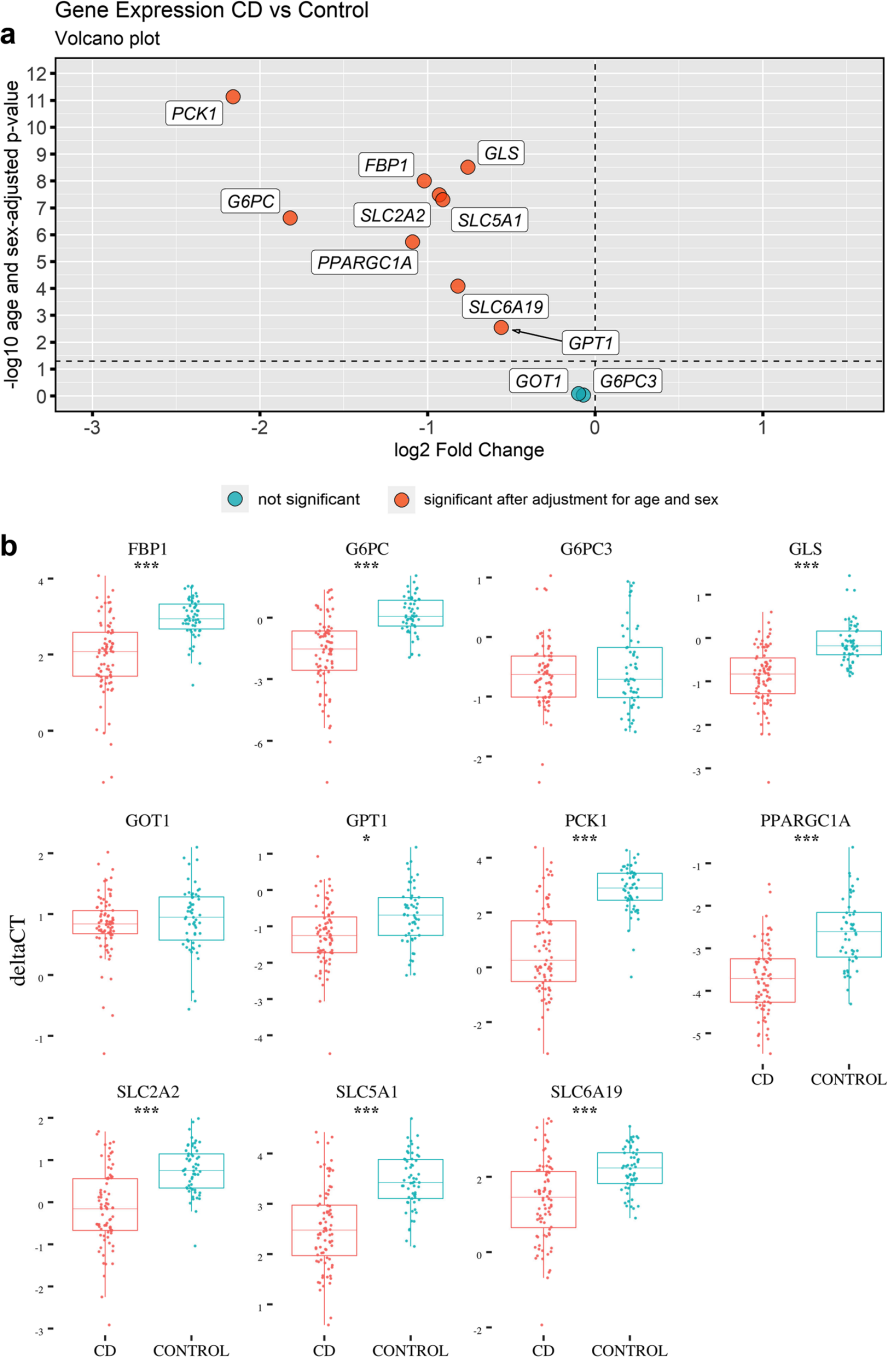
**a** Volcano plot showing the fold change on the *x*-axis and significance (−log *p*-value) on the *y*-axis. **b** Box plots of the gene expression levels in duodenal biopsies from children with untreated celiac disease (CD) (*n* = 84) and disease controls (CONTROL) (*n* = 58). All cases are visualized by individual dots. The middle horizontal line represents the median value, and the two “hinges” are the first and third quartiles. The “whiskers” extend to the minimum and maximum values within the 1.5 interquartile range of hinges. Significance of target genes marked with *p < 0.0045, **p < 0.00091, ***p < 0.000091 (Bonferroni-adjusted significance thresholds)

Analyzing gene expression levels in duodenal biopsies using qPCR, *FBP1*, *G6PC*, *GLS*, *GPT1*, *PCK1*, *PPARGC1A*, *SLC2A2*, *SLC5A1*, and *SLC6A 9* showed significantly lower expression in CD cases compared with controls, whereas *G6PC3* and *GOT1* showed no differences (Table 1 and Fig. 3). This remained significant, when adjusting for age and sex of study participants and multiple comparisons. Of the eight downregulated genes, *PCK1* was the most downregulated, with less than a quarter of the expression in the controls compared with cases (fold change = 0.22).

**Table 1:** Gene expression levels in duodenal biopsies from children with untreated celiac disease (CD) (*n* = 84) and disease controls (control) (*n* = 58) determined by qPCR

Gene	*p*-value	*p*-value (adjusted for age and sex)	CD, mean delta CT	Control, mean delta CT	delta delta CT	Fold change	% change
***FBP1***	**5.68E−11**	**9.96E−09**	1.95	2.96	− 1.02	0.49	− 103
***G6PC***	**1.24E−10**	**2.40E−07**	− 1.70	0.12	− 1.82	0.28	− 253
***G6PC3***	0.520	0.926	− 0.62	− 0.55	− 0.07	0.95	− 5
***GLS***	**7.02E−12**	**3.05E−09**	− 0.87	− 0.11	− 0.76	0.59	− 69
***GOT1***	0.249	0.823	0.82	0.93	− 0.10	0.93	− 7
***GPT1***	**2.08E−04**	**2.81E−03**	− 1.25	− 0.69	− 0.56	0.68	− 47
***PCK1***	**1.08E−16**	**7.27E−12**	0.66	2.82	− 2.16	0.22	− 347
***PPARGC1A***	**1.06E−11**	**1.84E−06**	− 3.72	− 2.63	− 1.09	0.47	− 113
***SLC2A2***	**1.38E−10**	**3.30E−08**	− 0.15	0.77	− 0.93	0.53	− 91
***SLC5A1***	**1.34E−11**	**4.95E−08**	2.53	3.44	− 0.91	0.53	− 88
***SLC6A19***	**5.16E−07**	**8.09E−05**	1.38	2.20	− 0.82	0.57	− 77

The Bonferroni-adjusted significance level for eleven target genes was 0.0045. Significant *p*-values for target genes are highlighted in bold. The far-right column shows the delta delta CT converted to percentage change in gene expression for CD patients compared to controls

### Correlation between gene expression levels and Marsh score and tTG antibody levels

Partial correlation analysis in the CD cases (Table 2) showed a strong correlation between the expression levels of the same nine genes and Marsh scores of patients. *PPARGC1A* also showed a significant correlation to IgA tTG antibody levels, while *FBP1*, *G6PC*, *GLS*, *GPT1*, *PCK1*, *PPARGC1A*, and *SLC5A1* showed a significant correlation to IgG tTG levels.

**Table 2:** Partial correlations analyzed in cases with CD, controlling for age

Gene	Marsh score		IgA-tTG		IgG-tTG	
	Correlation	***p***-value	Correlation	***p***-value	Correlation	***p***-value
***FBP1***	− 0.58	**1.51E−05**	− 0.19	0.19	− 0.43	**2.07E−03**
***G6PC***	− 0.47	**8.23E−04**	− 0.14	0.35	− 0.32	**0.03**
***G6PC3***	− 0.17	0.24	− 0.02	0.90	− 0.25	0.09
***GLS***	− 0.45	**1.23E−03**	− 0.18	0.23	− 0.38	**0.01**
***GOT1***	− 0.12	0.42	− 0.24	0.11	− 0.26	0.07
***GPT1***	− 0.57	**2.30E−05**	− 0.12	0.42	− 0.41	**3.54E−03**
***PCK1***	− 0.58	**1.67E−05**	− 0.26	0.08	− 0.36	**0.01**
***PPARGC1A***	− 0.39	**0.01**	− 0.40	**4.72E**− **03**	− 0.41	**3.48E−03**
***SLC2A2***	− 0.42	**3.35E−03**	− 0.10	0.49	− 0.22	0.13
***SLC5A1***	− 0.63	**2.03E−06**	− 0.13	0.36	− 0.30	**0.04**
***SLC6A19***	− 0.58	**1.74E−05**	− 0.19	0.18	− 0.26	0.07

Significant (two-tailed) correlations are shown in bold. Marsh scores were transformed into numbers: Marsh 2 = 1, Marsh 3A = 2, Marsh 3B = 3, and Marsh 3C = 4

*tTG* tissue transglutaminase

## Discussion

The present study shows that children with untreated CD have lower expression of genes involved in IGN in duodenal biopsies compared with children with a normal intestinal mucosa. Decreased expression is correlated to a higher Marsh score and, to a lesser degree, tTG antibody levels. These results suggest that CD patients could have an impaired function of IGN, either as a consequence of chronic intestinal inflammation in untreated disease or due to an impaired metabolic pathway shared with other chronic metabolic diseases suggested by the large number of traits shown to be associated with the *GLS*/*STAT1*/*STAT4* gene region. In the long-term perspective, a lower expression of these genes, which are central for IGN and thus important for metabolic homeostasis, may lead to other chronic diseases like NAFLD and diabetes if not recognized.

This study provides the first evidence of the downregulation of intestinal expression of *G6PC*, *GPT1*, *SLC6A19*, and *PPARGC1A* in CD. Glucose-6-phosphatase, the protein product of *G6PC*, has an essential role in gluconeogenesis by converting glucose-6-phosphate, which cannot be transported out of the cell, into glucose, which can then be released into the bloodstream, making its downregulation an especially important finding. Decreased expression of *PCK1*, *FBP1*, and solute carriers has previously been shown in adult CD patients but until now has not been put in the context of IGN [19–21]. Decreased expression of *GLS* is consistent with our previous finding of its downregulation in the GENEX material [1]. *G6PC3* and *GOT1* showed no significant changes in the expression; however, *G6PC* and *GPT1*, which catalyze the same reactions in IGN, are more critical for these respective functions [22, 23].

These data thus imply that the ability of the small intestine to perform gluconeogenesis and release glucose from the intestinal enterocyte might be severely decreased in patients with untreated CD. Decreased expression of *GLS*, *GPT1*, *PCK1*, *FBP1*, and *G6PC* suggest an impairment of the IGN pathway from the start of using glutamine in gluconeogenesis to releasing glucose into the blood. If only *GLS*, *GPT1*, and *PCK1* were downregulated, using glycerol, the second most important substrate of IGN [5] could still be possible since it enters gluconeogenesis in later steps, but since *FBP1* and *G6PC*, the protein products of which catalyze crucial final steps in gluconeogenesis, are also downregulated, it stands to reason that the whole pathway of IGN is impaired. We propose that the decreased expression of *PPARGC1A* might provide an explanation for this at a regulatory level since its protein has a key role in regulating hepatic gluconeogenesis. Even though it is unknown if it has a similar role in the intestine, it does not seem entirely implausible. Decreased expression of *SLC6A19* suggests the ability to absorb glutamine, as well as other neutral amino acids transported by the SLC6A19 protein (also known as B0AT1), from the intestinal lumen is impaired. Lower expression of *SLC5A1* (SGLT1) and *SLC2A2* (GLUT2*)* indicate decreased capacity for glucose transport.

The metabolic effects of impaired IGN in humans are not entirely clear. Studies on IGN in animal models show that an increase in glucose levels in the portal vein provides signals that increase satiety and improve energy homeostasis. Induced high levels of IGN appear to offer protection against metabolic disease, while impairment leads to signs of dysregulated glucose control and hepatic steatosis [7, 10].

CD patients are at increased risk of NAFLD, with the highest risk seen during the first years after diagnosis and the largest relative risk increase seen in patients with a normal BMI [24, 25]. We speculate that impaired IGN could provide an explanation for the increased risk of NAFLD in CD patients. Our study does not examine whether expression of IGN-related genes return to normal in CD patients treated with a gluten-free diet, but the correlation with Marsh scores suggests a lower degree of inflammation might improve IGN. If IGN is normalized when CD is treated, perhaps this could be part of the explanation for why the risk of NAFLD is at its highest in the first year after CD diagnosis, when the gluconeogenetic capability of the intestine perhaps has not fully recovered. Such a recovery might also be suggested by a 1968 study of glutaminase enzymatic activity, which found lower levels in untreated CD patients that seemed to recover in patients on treatment with a gluten-free diet [26]. Studying the expression of IGN-related genes in patients before and after treatment would be an important next step.

This study has several limitations. The expression of the selected genes in CD cases were compared with disease controls, i.e., these were children referred for an upper endoscopy investigated for other intestinal diseases affecting the gut. It cannot be excluded that the disease controls may have had conditions that can affect the expression of the selected genes. However, all disease controls had normal mucosal findings, and children with inflammatory bowel disease and *Helicobacter pylori* infections were excluded prior to analysis. Another limitation was that cases and controls were not age- and sex-matched. Still, when adjusting for age and sex, the results remained significant. The strength of the study is that children were enrolled from four sites by pediatric gastroenterologists with long clinical experience in diagnosing and treating children with CD. Enrollment of study participants occurred in 2012 or earlier, e.g., when the intestinal biopsy was the golden standard for diagnosis of CD, meaning that also children with very high levels of IgA-tTG were included in the cohort. Moreover, we previously had all intestinal biopsies reviewed and scored histologically by a single pathologist blinded to the clinical and serological data before the analysis to reduce observation bias and potential risk of discordant classification of cases and controls between the sites.

The results from the present study raise several questions. It is not clear whether the downregulation of the target genes is specific for CD or related to intestinal inflammation in general. The association between the gene region containing *GLS* and other autoimmune traits, many of which show an increased risk of metabolic disease, could suggest that the downregulation of IGN could also be present in other inflammatory diseases. Thus, further studies of IGN in other diseases are warranted. Also, the study does not answer if impaired IGN is involved in the risk of developing the disease or if it is a response to other disease-initiating mechanisms in CD. In addition, we have not explored if changes in the microbiota could possibly have an effect on the gene expression or if the IGN expression is affected by body mass index and glucose levels. Moreover, while we see a significant correlation between decreased IGN gene expression and the degree of damage in the mucosa, the study does not answer if treatment with a gluten-free diet leading to the healing of the intestinal mucosa restores the expression of genes involved in IGN. Furthermore, it could be of interest to explore whether these genes are downregulated in other intestinal mucosal diseases such as immune deficiency disorders or autoimmune enteropathy.

## Conclusion

In conclusion, children with untreated CD show downregulation of genes critical for IGN in the intestinal mucosa, suggesting impairment of IGN. An impaired IGN may explain the increased risk of metabolic diseases like NAFLD found in CD patients as adults. Further studies around IGN and its role both in CD and other diseases are warranted.

## Supplementary Information

**Additional file 1: Table S1.** Traits listed in the GWAS Catalog for the GLS/STAT1/STAT4 region (https://www.ebi.ac.uk/gwas) and the most associated SNP reported for each trait including their reported eQTLs* in the Genotype-Tissue Expression (GTEx) portal (https://www.gtexportal.org).

**Additional file 2: Table S2.** Demographic table summarizing patient characteristics.

## Data Availability

Datasets from the study are available from the corresponding author upon reasonable request.
