# Supplementary material for: Intestinal gluconeogenesis is downregulated in pediatric patients with celiac disease
Source: BMC Med. 2022 Nov 11;20:440. doi: 10.1186/s12916-022-02635-3 (PMC9652951; doi:10.1186/s12916-022-02635-3)
Supplement: Supplementary file 1 — Additional file 1: Table S1. Traits listed in the GWAS Catalog for the GLS/STAT1/STAT4 region (https://www.ebi.ac.uk/gwas) and the most associated SNP reported for each trait including their reported eQTLs* in the Genotype-Tissue Expression (GTEx) portal (https://www.gtexportal.org). [file 12916_2022_2635_MOESM1_ESM.pdf]

Table S1. Traits listed in the GWAS Catalog for the GLS/STAT1/STAT4 region (https://www.ebi.ac.uk/gwas) and the most associated SNP reported for each trait including their reported eQTLs* in the Genotype-Tissue Expression (GTEx) portal (https://www.gtexportal.org).

Trait	Study accession	Variant and risk allele	Chr	Location (bp)	P-value	Mapped gene	eQTL gene	tissue
Systemic lupus erythematosus (SLE)	GCST011956	rs11889341-T, rs7568275-?	2	191079016	5.00E-123	STAT4	GLS	Colon - Transverse
Oral ulcer	GCST007839	rs11684030-A	2	191152153	1.00E-42	STAT4, HMGB1P27	STAT4	Esophagus - Muscularis
Systemic scleroderma, rheumatoid arthritis, myositis, SLE	GCST007278	rs10174238-?	2	191108308	3.00E-42	STAT4		
Thyroid preparation use measurement	GCST007932	rs11889341-T	2	191079016	3.00E-39	STAT4		
Hypothyroidism	GCST007073	rs7582694-?	2	191105394	3.00E-39	STAT4		
Autoimmune thyroid disease	GCST010571	rs7568275-G	2	191101726	5.00E-39	STAT4	GLS	Colon - Transverse
Mean platelet volume	GCST90002346	rs1921911-C	2	190855644	6.00E-31	GLS	GLS	Muscle - Skeletal
Rheumatoid arthritis	GCST90013534	rs11889341-T	2	191079016	4.00E-30	STAT4		
Reticulocyte count	GCST90002385	rs398105120-AT	2	190944197	4.00E-25	STAT1, GLS		
Primary biliary cirrhosis	GCST005581	rs7574865-C, rs3024921-T	2	191099907	9.00E-25	STAT4		
Reticulocyte measurement	GCST90002387	rs11687659-A	2	190876471	6.00E-24	GLS	GLS	Muscle - Skeletal
Autoimmune disease	GCST007071	rs7568275-?	2	191101726	2.00E-24	STAT4	GLS	Colon - Transverse
Body height	GCST007841	rs35917062-?	2	190796102	9.00E-23	GLS		
Systemic scleroderma	GCST009131	rs3821236-A	2	191038032	2.00E-23	STAT4		
Juvenile idiopathic arthritis	GCST90010715	rs11889341-T	2	191079016	2.00E-19	STAT4		
Sjogren syndrome	GCST002217	rs10168266-T	2	191071078	2.00E-17	STAT4		
Inflammatory bowel disease	GCST003043	rs1517352-C	2	191066738	4.00E-14	STAT4		
Systemic, polyarticular and oligoarticular juvenile idiopathic arthritis, rheumatoid factor negative	GCST005528	rs10174238-G	2	191108308	1.00E-13	STAT4		
Multiple sclerosis	GCST009597	rs6738544-C	2	191124630	2.00E-13	STAT4		
Limited scleroderma	GCST005555	rs10174238-?	2	191108308	1.00E-11	STAT4		
Systemic scleroderma, SLE	GCST002069	rs7601754-?	2	191075725	3.00E-11	STAT4		
Rheumatoid arthritis, celiac disease	GCST008644	rs6749371-?	2	191037458	7.00E-11	STAT4		
PHF-tau measurement	GCST010340	rs7033192-? x rs6434425-?	2	190857590	6.00E-10	SLC8A1 - GLS		
Birth weight	GCST009437	rs1547550-C	2	190980999	1.00E-10	STAT1	GLS/STAT4	Muscle - Skeletal/Nerve - Tibial

Mean corpuscular hemoglobin	GCST007068	rs7565237-?	2	190971964	3.00E-10	STAT1	GLS	Muscle - Skeletal
Crohn's disease	GCST003044	rs1517352-C	2	191066738	1.00E-10	STAT4		
Hepatocellular carcinoma	GCST001775	rs7574865-G	2	191099907	2.00E-10	STAT4		
Immune system disease	GCST000987	rs7574865-T	2	191099907	4.00E-10	STAT4		
Non-typhoidal Salmonella bacteremia	GCST005590	rs13390936-T	2	191090090	9.00E-10	STAT4		
BMI-adjusted waist-hip ratio	GCST009858	rs78219026-?	2	190881507	3.00E-09	GLS	GLS	Artery - Aorta
Waist-hip ratio	GCST007067	rs6434426-?	2	190861883	7.00E-09	GLS	GLS	Artery - Tibial
Neutrophil percentage of granulocytes	GCST004623	rs60976990-T	2	190991701	2.00E-09	STAT1		
Behcet's syndrome	GCST001804	rs7574070-A	2	191145762	1.00E-09	STAT4	STAT4	Cells - EBV-transformed lymphocytes
Anti-centromere-antibody-positive systemic scleroderma	GCST005552	rs10174238-?	2	191108308	2.00E-09	STAT4		
Ulcerative colitis	GCST003045	rs1517352-C	2	191066738	2.00E-09	STAT4		
Eosinophil count	GCST90002381	rs6752770-G	2	191108837	2.00E-09	STAT4		
Type I diabetes mellitus	GCST90013445	rs7582694-G	2	191105394	3.00E-09	STAT4		
Celiac disease	GCST009874	rs6749371-?	2	191037458	7.00E-09	STAT4		
Self reported educational attainment	GCST006442	rs66721975-A	2	190836459	2.00E-08	GPR39, GLS		
Protein measurement	GCST011427	rs2066799-T	2	190986759	3.00E-08	STAT1		
JT interval, response to sulfonylurea	GCST004032	rs12468579-?	2	190967538	5.00E-08	STAT1	GLS	Muscle - Skeletal
Amyloid-beta measurement	GCST010339	rs12065191-? x rs3024861-?	2	191059880	8.00E-08	STAT4		
Rheumatoid arthritis, Crohn's disease	GCST90016610	rs12612769-?	2	191089272	2.00E-07	STAT4		
Rheumatoid arthritis, ulcerative colitis	GCST90016625	rs11889341-?	2	191079016	2.00E-07	STAT4		
Vitiligo	GCST004785	rs199559999-?	2	191090480	3.00E-07	STAT4		
Longevity	GCST009448	rs13033350-A	2	190826756	1.00E-06	GLS	GLS	Muscle - Skeletal
Diabetes mellitus, coronary artery disease	GCST90014128	rs148894474-C	2	190815604	3.00E-06	GLS		
Threonine measurement	GCST009391	rs1607187-?	2	190950312	3.00E-06	GLS, STAT1	GLS	Muscle - Skeletal
Biliary liver cirrhosis	GCST001685	rs7574865-T	2	191099907	1.00E-06	STAT4		
Myositis	GCST006051	rs4853540-?	2	191052591	2.00E-06	STAT4		

*eQTL = expression Quantitative Trait
