# Supplementary material for: Intestinal gluconeogenesis is downregulated in pediatric patients with celiac disease
Source: BMC Med. 2022 Nov 11;20:440. doi: 10.1186/s12916-022-02635-3 (PMC9652951; doi:10.1186/s12916-022-02635-3)
Supplement: Supplementary file 2 — Additional file 2: Table S2. Demographic table summarizing patient characteristics. [file 12916_2022_2635_MOESM2_ESM.pdf]

**Table S2.** Demographic table summarizing patient characteristics.

| Diagnosis | number | percent female | Age (mean ± SD) |
|---|---|---|---|
| CD | 84 | 70% | 6.1 ± 3.7 *** |
| Disease controls | 58 | 67% | 11.6 ± 4.5*** |
| ***Controls per diagnosis*** | | | |
| Food Intolerance | 4 | 100% | 7.8 ± 5.5 |
| Eosinophil Esophagitis | 1 | 0% | 13.1 ± 0 |
| Gastritis | 11 | 54% | 11.2 ± 3.8 |
| GERD | 24 | 62% | 10.2 ± 4.2 |
| RAP | 9 | 78% | 12.9 ± 4 |
| Family history of CD | 2 | 0% | 2.6 ± 1.2 |
| Family history of UC | 1 | 100% | 16.5 ± 1.9 |
| Hyper-IgD syndrome | 1 | 0% | 14.8 ± 0 |
| IBS | 1 | 0% | 4.5 ± 0 |
| IgA deficiency | 1 | 0% | 16.8 ± 0 |
| Malaborption syndrome | 1 | 0% | 9.5 ± 0 |
| Obstipation | 2 | 100% | 11.2 ± 1.3 |
| RAP & T1D | 1 | 0% | 16.8 ± 0 |
| GERD & T1D | 1 | 100% | 18.3 ± 0 |

CD = Coeliac Disease, GERD = Gastroesophageal reflux disease, RAP = Recurrent abdominal pain, IBS = Irritable bowel syndrome, UC = Ulcerative Colitis, IGD = Immunoglobin deficiency, T1D, type 1 diabetes, SD = standard deviation. *** $p < 0.001$
